# Giant stone adhered in the vaginal cavity in a female patient with type 2 diabetes mellitus: a case report

**DOI:** 10.1186/s13256-024-04355-z

**Published:** 2024-03-17

**Authors:** César Alas-Pineda, Kristhel Gaitán-Zambrano, Génesis Sarahí Chávez Paredes, Gloria Cárcamo-Portillo, Hilda A. Argeñal-Guifarro, Luis Zúñiga-Girón

**Affiliations:** 1 Departamento de Medicina Interna, Hospital Dr. Mario Catarino Rivas, San Pedro Sula, Honduras; 2 Facultad de Medicina y Cirugía, Universidad Católica de Honduras - Campus San Pedro y San Pablo, San Pedro Sula, Cortés, Honduras; 3 Departamento de Ginecología y Obstetricia, Hospital Dr. Mario Catarino Rivas, San Pedro Sula, Honduras; 4 Médico Especialista en Ginecología y Obstetricia, Hospital Dr. Mario Catarino Rivas, San Pedro Sula, Honduras

**Keywords:** Cystolithotomy, Diabetes mellitus, Vesicovaginal fistula, Bladder stones, Giant stone, Vaginal cavity

## Abstract

**Background:**

Bladder lithiasis comprises 5% of urological lithiasis. Large bladder stones associated with vesicovaginal fistulas are rare, and the risk factors are not an isolated process. There are metabolic comorbidities associated with this pathology, including diabetes mellitus.

**Case presentation:**

A 70-year-old Mestizo patient is presented, reporting dysuria, pollakiuria, and abdominal pain of 4 months of evolution, located in the hypogastric region, also with a sensation of a foreign body in the vaginal introitus. In her pathological history, she presented type 2 diabetes mellitus. A computed tomography scan of the abdomen and pelvis was performed, reporting a tumor lesion in the abdominal wall. Therefore, surgical intervention was performed by cystolithotomy, obtaining a giant stone adhered to the vaginal wall with a size of 10 cm × 12 cm.

**Conclusion:**

Early detection of this pathology should be exhaustive in patients with characteristics and comorbidities associated with stone development to avoid possible complications, such as vesicovaginal fistulas.

## Background

Urolithiasis is a pathology characterized by the production of stones in the urinary system, primarily in the kidney (nephrolithiasis), ureters, bladder, and/or urethra, resulting from one or more alterations in urinary composition that favor urine crystallization [1]. Currently, vesical lithiasis constitutes 5% of urological lithiasis cases, affecting both men and women, although it is traditionally considered to predominate in the male gender among age groups between the third and sixth decades of life [2–4]. The presence of bladder stones is on the rise due to demographic, dietary, and genetic factors contributing to the development of this condition, such as metabolic syndrome, hypertension, and diabetes mellitus [1, 5]. Additionally, large bladder stones associated with vesicovaginal fistulas are relatively rare, and risk factors are often interconnected, such as: chronic urinary retention, bladder outlet obstruction, urinary tract infection, prolonged catheterization, presence of a foreign body, neurogenic bladder, and even rare cases involving a bladder stone around an incorporated arterial graft [6–8].

A vesicovaginal fistula is an abnormal communication between the bladder and the vagina, being the most common type among urogenital fistulas [8–10]. Its etiology varies based on its condition: congenital or acquired [9]. Circumstances, such as socioeconomic status, early motherhood, and inadequate obstetric care, have been shown to promote vesicovaginal fistula formation [8, 9]. Typically, in developing countries, vesicovaginal fistula occurs following obstetric trauma or is iatrogenic [11]. Patients with bladder stones adhered to the vaginal cavity report a range of clinical manifestations secondary to the inflammation generated by these alterations in the bladder mucosa, often seeking medical attention due to lower urinary tract symptoms or hematuria [3, 4, 12].

The frequency of vesicovaginal fistula is low in the developed world [4, 9]. However, in developing regions, it has a much higher prevalence. It has been estimated that at least 3 million women in low socioeconomic countries suffer from unrepaired vesicovaginal fistulas, with approximately 30,000 to 130,000 new cases each year due to obstructed labor in Africa alone [9]. Nevertheless, the true incidence, primary etiology, and prevalence of vesicovaginal fistula in women worldwide are difficult to determine, as the precise relationship between stone formation and vesicovaginal fistula remains unknown.

Bladder stones of considerable size adhered to the vaginal wall are exceedingly rare [4]. Nonetheless, early detection of this condition must be meticulous in patients with associated characteristics and comorbidities predisposing them to stone development to prevent potential complications. The stone can exert pressure on the bladder wall, causing tissue necrosis and, subsequently, vesicovaginal fistula [4, 9]. Therefore, this study reports the first case of a massive bladder stone in the vaginal cavity, measuring 10 cm × 12 cm, in an elderly female in Honduras.

## Case presentation

**Fig. 1:**
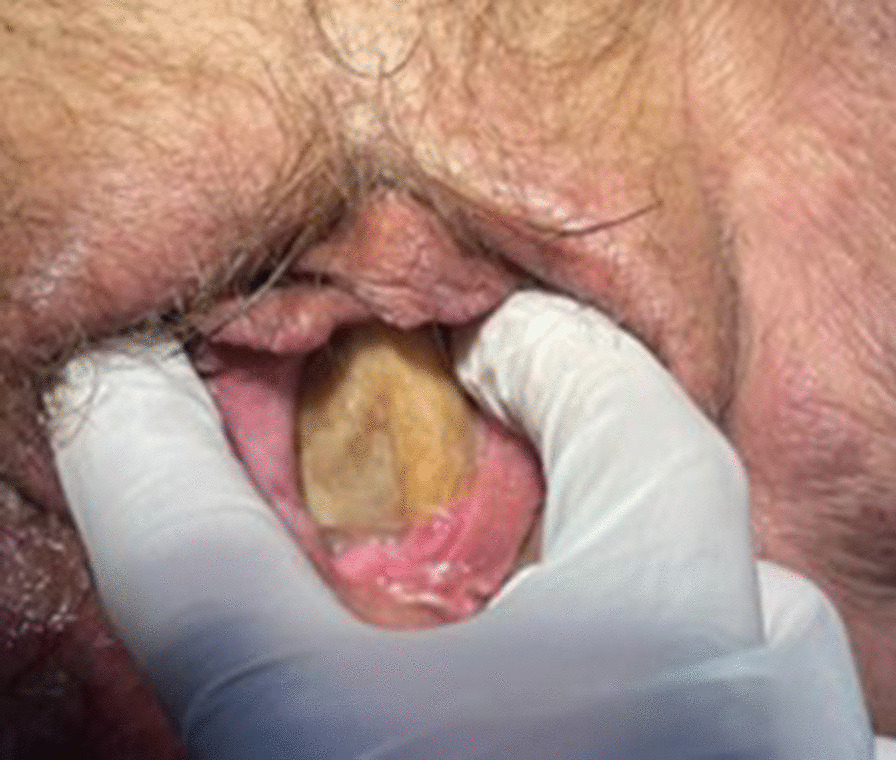
Obstructive mass at the vaginal introitus

A 70-year-old Mestizo female patient presented to the hospital with a 4-month history of dysuria, polyuria, and lower abdominal pain. The pain was colicky, localized in the hypogastric region, and radiating to the suprapubic region without aggravating or alleviating factors. She also reported a sensation of a foreign body in the vaginal introitus. Her medical history included a diagnosis of type 2 diabetes mellitus 6 months prior, with good adherence to treatment. Surgical history revealed hernioplasty performed 12 years ago, and gynecological history indicated eight pregnancies and eight deliveries. Physical examination revealed a mobile, firm, and tender mass in the left periumbilical area. In the lithotomy position, examination of the external genitalia revealed a moist vulva with urinary remnants, mild hyperemic inflammatory changes, and an evident obstructive mass at the vaginal introitus (Fig. 1), suggestive of a giant calyceal-type calculus.

**Fig. 2:**
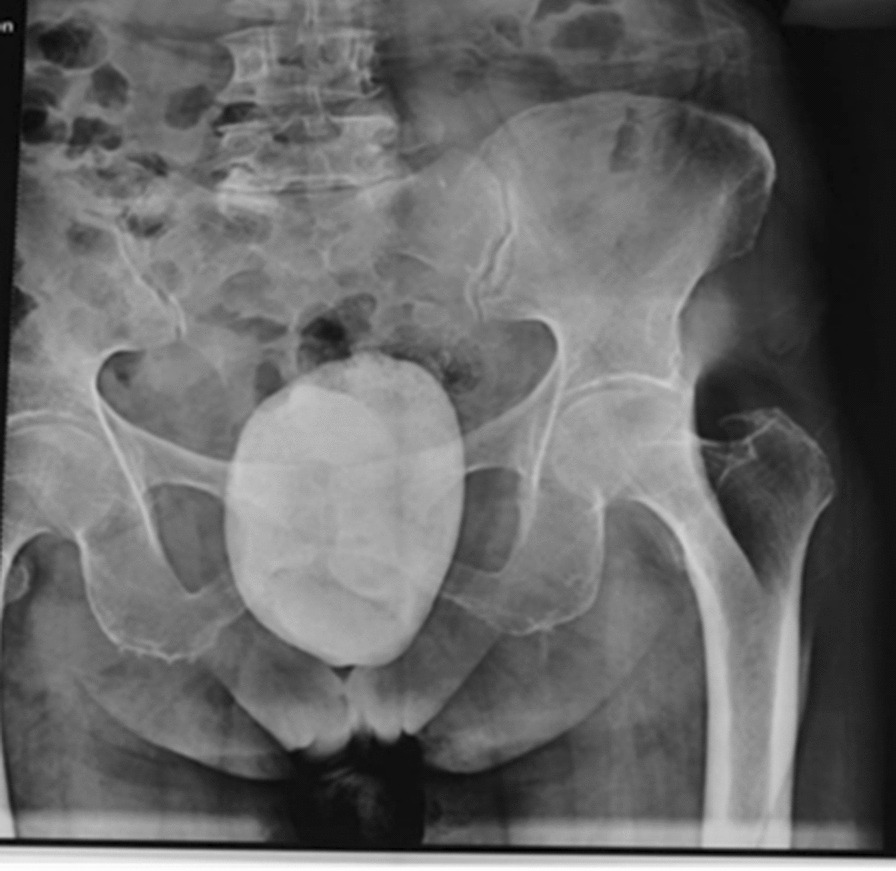
Anteroposterior pelvic radiograph showing a radio-opaque ovoid mass in the lower pelvis, suggestive of a bladder stone

**Fig. 3:**
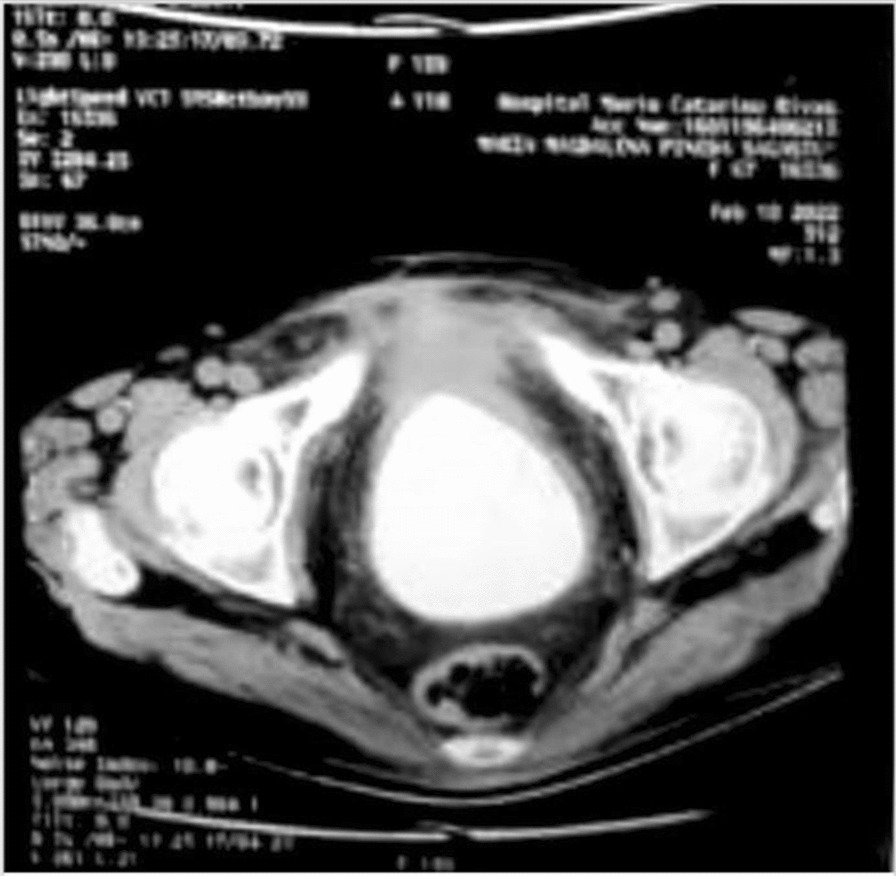
Plain computed tomography scan at the pelvic level, depicting a hyperdense ovoid mass located within the urinary bladder, consistent with a bladder stone

A plain pelvic radiograph reported a large stone-like image projecting in the pelvic region, raising the possibility of a giant bladder stone (Fig. 2). Subsequently, an abdominopelvic computed tomography (CT) scan revealed a solid tumor-like lesion with significant calcification involving the fat and muscle planes of the lower abdominal wall. Additionally, a giant calculus, measuring approximately 10 cm × 12 cm, was observed; however, its precise anatomical location could not be determined, and it was suspected to be in either the bladder or cervix (Fig. 3). The patient also exhibited right renal lithiasis and simple cortical cysts bilaterally, with the presence of an extrarenal pelvis. Urinalysis indicated a pH of 7.5, abundant bacteria, numerous erythrocytes, moderate epithelial cells, and few renal cells, with a positive urine culture for *Escherichia coli*. A routine complete blood count showed no significant findings.

**Fig. 4:**
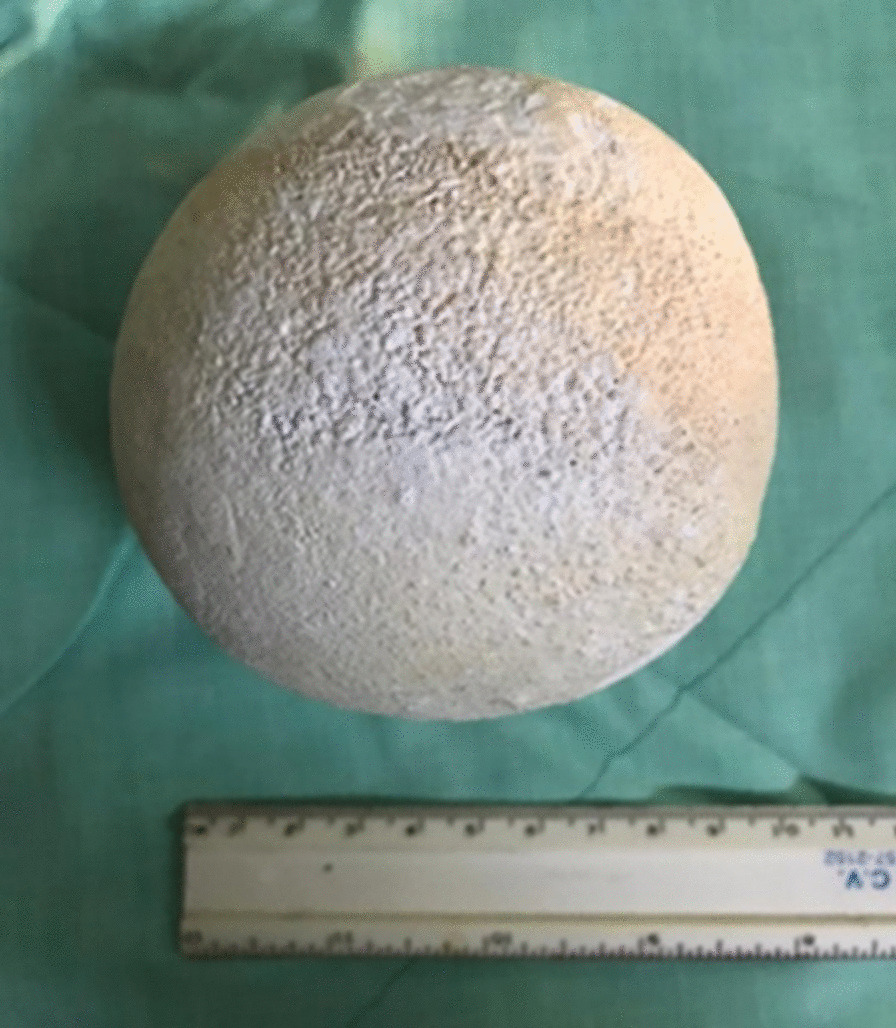
Giant calculus with regular borders, measuring 10 cm × 12 cm in size

**Fig. 5:**
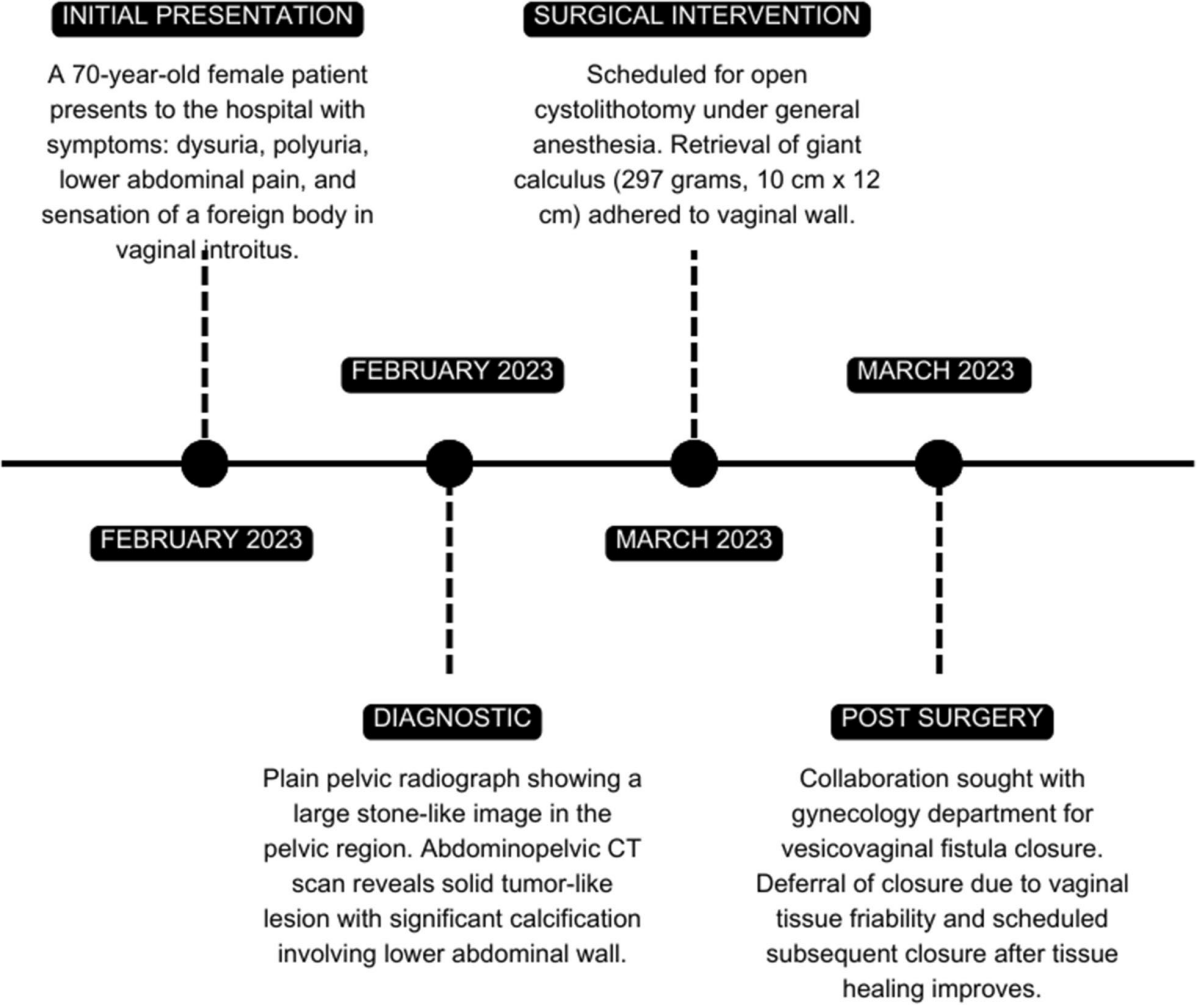
Timeline course

The patient was referred to the urology department, and surgical intervention was scheduled for open cystolithotomy under general anesthesia. An incision in the lower abdominal wall was performed to access the bladder. A giant calculus adhered to the vaginal wall was retrieved, weighing 297 g with dimensions of 10 cm × 12 cm (Fig. 4). Biochemical analysis of the giant calculus revealed the presence of calcium oxalate and urate. Bladder repair was performed, and collaboration with the gynecology department was sought for vesicovaginal fistula closure, which was deferred due to vaginal tissue friability. Subsequent surgical closure of the vesicovaginal fistula was scheduled once tissue healing had improved. The postoperative course was uneventful; follow-up visits continue at regular intervals to assess bladder function and healing (Fig. 5).

## Discussion

Urolithiasis accounts for 5% of urinary tract calculi, with a prevalence varying from 1% to 20% in the general population, and recurrence rates can exceed 50% depending on geographical, climatic, ethnic, dietary, and genetic factors [5, 13]. Medlen *et al*. classified vesicovaginal fistulas according to their etiology: congenital and acquired (whether or not they are associated with other pathologies) [9]. In the former case, they are typically associated with other urogenital malformations. In the latter, they can be categorized as obstetric, malignant, radiation induced, or miscellaneous [9]. The patient’s history of eight previous vaginal deliveries increased the likelihood of unnoticed gynecological-obstetric trauma. Furthermore, a past hernioplasty may have potentially predisposed her to the formation of the vesicovaginal fistula, which was subsequently evident during the surgical intervention.

The most common cause (> 75%) of vesicovaginal fistula, secondary to vesical lithiasis, is gynecological surgery, such as obstructed labor and bladder wall necrosis due to pressure [4, 9, 11]. Currently, there are no reported data regarding the incidence of this condition in Honduras. Additionally, factors associated with vesical lithiasis, such as diet and lifestyle, lead to its occurrence in younger patients, especially in those with a history of obesity and/or diabetes mellitus [14, 15]. It is worth noting that the diagnosis of diabetes mellitus aligns with the findings in this case. On the other hand, it is known that supersaturation and crystallization are the main drivers of the etiopathogenesis of uric acid, xanthine, and cystine calculi, but these physicochemical concepts do not adequately explain the formation of calcium-based nephrolithiasis [1, 7]. In our case, the calculus was composed of calcium oxalate and urate (not disclosed until analysis).

Typically, vesical lithiasis presents as filling pain (30–60%), urgency (40–50%), and terminal hematuria, although there are documented cases in which it may lead to complications, such as vesicocutaneous fistula and pelvic pain [4, 5, 16]. In the presence of a fistula, the main symptoms are dysuria and continuous incontinence [4]. While various clinical manifestations may be associated with this condition, our patient experienced symptoms consistent with vesicovaginal fistula, as previously described by Francisca *et al*. [4].

Presumptive diagnosis was obtained through a plain pelvic radiograph, which was subsequently confirmed by an abdominopelvic computed tomography (CT) scan, as recommended by Cicione *et al*. [3]. While X-rays can reveal the presence of bladder stones, uric acid stones may not be visible, warranting a CT scan if economically feasible [3]. It is also advisable to request urine cultures, as patients with bladder stones adhered to the vaginal cavity often experience recurrent urinary infections. Bacteria promote lithogenesis, precipitating urinary infection development [17]. A urine culture was performed on the patient, yielding a positive result, in line with prior literature.

The treatment of vesical lithiasis associated with vesicovaginal fistula depends on the size, location, and surgeon’s expertise [4, 11, 18]. Several studies have concluded that multiple treatment alternatives exist based on stone size. For instance, when the stone exceeds 3 cm, the treatment of choice is cystolithotomy [4, 11, 12]. In our patient, open cystolithotomy was performed, as the stone exceeded a diameter of 10 cm. Conversely, Bahilo *et al*. [19] demonstrated that extracorporeal shock wave lithotripsy (ESWL) is a minimally invasive treatment for vesical lithiasis. When performed with proper technique, ESWL achieves high clinical and economic effectiveness, in line with the findings of Padron *et al*. [20].

No complications were observed following surgical management in our experience, and the patient underwent a two-stage surgical approach, as suggested by Francisca *et al*. [4]. The patient left the operating room in stable condition, with no recurrence noted during follow-up through outpatient consultations.

## Conclusion

Presently, Honduras lacks statistical data to determine the incidence of giant lithiasis associated with the vaginal wall. This study presents the first reported case of a giant vesical calculus associated with a vesicovaginal fistula, measuring 10 cm × 12 cm, in an elderly female in Honduras. Large vesical stones are, indeed, rare in the global female population. It is essential to consider patients with comorbidities and surgical histories for early detection of growing calculi, as early intervention can prevent the development of complications such as vesicovaginal fistula. The treatment of choice in this case was open surgery (cystolithotomy), and optimal results were achieved, with the patient leaving the operating room in stable condition.

## Data Availability

Patient’s files and datasets used to support the findings of this study are restricted to protect the privacy of clinical data. Data are available to investigators who comply the criteria for access to confidential data under request. Requests for access to these data should be directed to César Alas‑Pineda: cesar_alas10@hotmail.com.

## Abbreviations

CT: Computed tomography
ESWL: Extracorporeal shock wave lithotripsy
